# Influence of Hydrolysis Degree and Molecular Weight on the Structure and Absorption Properties of Polyvinyl Alcohol Freeze-Dried Porous Polymer

**DOI:** 10.3390/bioengineering13030259

**Published:** 2026-02-24

**Authors:** Ming Tian, Chaoqiao Zhu, Qingfeng Yang, Simin Fan, Jinkai Pang, Le Liu, Debao Wang, Dequan Zhang, Xin Li, Chengli Hou

**Affiliations:** 1 College of Food Science and Engineering, Shanxi Agricultural University, Taiyuan 030000, China; 13080204653@163.com (M.T.);; 2 Institute of Food Science and Technology, Chinese Academy of Agricultural Sciences, Key Laboratory of Agro-Products Quality and Safety Control in Storage and Transport Process, Ministry of Agriculture and Rural Affairs, Beijing 100193, China; yangqingfeng@caas.cn (Q.Y.); fan2019simin@163.com (S.F.); 15628639521@163.com (J.P.); liule1017@126.com (L.L.); wangdebao@caas.cn (D.W.); zhang0118@126.com (D.Z.); xinli.caas@gmail.com (X.L.)

**Keywords:** hydrogen bonding, cross-linking, super absorbency freeze-dried porous polymer

## Abstract

The water absorption of polyvinyl alcohol (PVA) freeze-dried porous polymer is critically influenced by its molecular structure. The hydrolysis degree and molecular weight of PVA were identified as key factors in the design of freeze-dried porous polymers for enhanced structure and stability. The complex interactions between water absorption and structural characteristics in freeze-dried porous polymers were investigated. This was achieved by varying the degree of hydrolysis and molecular weight of the PVA. The results indicate that as the degree of PVA hydrolysis increases, the water absorption and structural stability of the freeze-dried porous polymer are significantly improved. These performance enhancements are attributed to the synergistic effects of hydrogen bonding interactions and molecular chain entanglement between PVA-sodium polyacrylate (PAAS) chains and PVA-PVA chains, collectively forming a denser and more stable three-dimensional network structure. Additionally, the incorporation of high molecular weight PVA significantly reduced the water absorption capacity of the freeze-dried porous polymer. However, freeze-dried porous polymers prepared using low molecular weight polyvinyl alcohol exhibit poor structural stability. Specifically, when the PVA molecular weight is 7200-8100, and the degree of hydrolysis is 99%, the freeze-dried porous polymer exhibits a maximum porosity of 92%, a density of 82 mg/cm^3^, and a water absorption capacity of 38 g/g. Overall, this work provides the theoretical basis and technical support for its application in absorbent pads.

## 1. Introduction

During storage and sales, fresh agricultural products, such as meat and aquatic products, lose juice due to dehydration of muscle tissue and damage to myofibrils [1]. The water vapor generated by transpiration and respiration in fruits and vegetables condenses into droplets, which eventually accumulate at the bottom of the packaging [2]. These phenomena contribute to the deterioration of quality and cause undesirable economic losses. Currently, absorbent pads are widely employed to absorb lost juices [3], ensuring product quality and optimizing consumer experience. Creating a relatively dry environment inside the packaging prevents the growth of microbes, preserves the sensory quality of the food, and extends its shelf life [4]. Absorbent pad materials are usually made up of polymer films (such as PE, PVC, and PET) and nonwoven materials [5,6]. However, natural polymers frequently exhibit insufficient stability and water uptake, while synthetic polymers, conversely, present the persistent issue of intrinsic non-degradability [7]. With increasing government focus on environmental sustainability, the potential for microplastic pollution from their disposal has become a new focus of environmental attention, limiting the widespread use of these products. Furthermore, the materials currently used in absorbent pads still have limitations, including insufficient absorbency, complex manufacturing processes, and the use of non-biodegradable raw materials [8]. Therefore, the development of new, highly absorbent, biodegradable pad materials has become the current focus of industrial research and development.

Freeze-dried porous polymers are a class of materials typically produced by lyophilization. This process involves the sublimation of ice under vacuum, which not only dehydrates the material but also creates an interconnected porous structure in the final product [9,10]. Its unique three-dimensional structure gives it excellent water absorption and retention properties, making it suitable for use as an oil/water separation application [11], facial mask base material [12], and absorbent pad [13,14]. Biodegradable and biocompatible cellulose, polysaccharides, and biopolymers have received extensive attention in the preparation of freeze-dried porous polymer. PAAS is a superabsorbent polymer capable of absorbing hundreds of times its own mass in water [15]. These characteristics make it an ideal candidate for advanced adsorption materials. The independent application of this material is hindered by its lack of water stability and structural integrity, despite its exceptionally high-water absorption capacity. To overcome this limitation, it is commonly blended with other polymers to enhance its properties [16]. Among these, polyvinyl alcohol (PVA) is valued for its role in improving the mechanical strength and physical properties [17]. Previous reports indicate that during preparation, PVA readily engages in hydrogen bonding owing to its abundant hydroxyl groups. It forms network structures with biopolymers such as polysaccharides (chitosan [18], sodium alginate [19]), starch [20] and cellulose wood fibers [21]). It provides the material with excellent mechanical properties and structural stability.

PVA is a semi-crystalline polymer derived from polyvinyl acetate. It is characterized by several biological advantages, including water solubility, non-toxicity, biodegradability, and biocompatibility [22,23]. The properties and applications of PVA are governed mainly by its molecular weight and the degree of hydrolysis [24]. The abundance of free hydroxyl groups in PVA is determined by its degree of hydrolysis and the content of residual acetate groups. This abundance, in turn, governs the polymer’s characteristic chemical properties, solubility, and crystallizability [25,26]. It is the high compatibility and synergistic interactions between the materials that enable them to form a stable structure. Cross-linking PVA with PAAS contributes to enhanced structural stability. However, the precise control of hydrolysis degree and molecular weight, required to synergistically optimize stability and microstructure, remains a core research challenge.

This study utilized PVA with varying degrees of hydrolysis and molecular weights to optimize the structural stability of the PAAS freeze-dried porous polymer. Porous freeze-dried porous polymer materials were prepared via freeze-drying. With the molecular weight held constant, the incompletely hydrolyzed PVA was compared with the partially hydrolyzed PVA. With a fixed degree of hydrolysis, the molecular weight of the PVA varied to regulate the hydrogen bond density and network structure of the PVA-PAAS freeze-dried porous polymer, thereby influencing their water absorption capacity (WAC) and swelling behavior. The physical and chemical structural properties of the freeze-dried porous polymer were investigated. This work will provide key support for optimizing the stability and comprehensive performance of freeze-dried porous polymer materials.

## 2. Materials and Methods

### 2.1. Materials

PVA samples with a fixed molecular weight range of 72,000~81,000 but varying degrees of hydrolysis (87~89%, 96~98%, and 98~99%), along with samples at a fixed degree of hydrolysis of 98~99% but different molecular weights (31,000~50,000 and 190,000), were obtained from Macklin Bio-Technology Co., Ltd. (Shanghai, China). PAAS was purchased from Yuanye Bio-Technology Co., Ltd. (Shanghai, China). Other reagents (analytically pure) were purchased from Sinopharm Chemical Reagent Co., Ltd. (Beijing, China).

### 2.2. Preparation and Characterization of Freeze-Dried Porous Polymer

**Figure 1:**
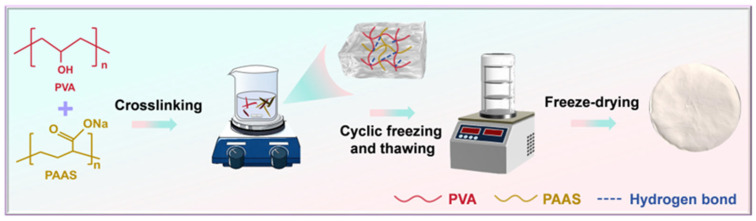
Flowchart for preparing freeze-dried porous polymer with different degrees of hydrolysis and molecular weights via freeze-drying.

The PVA-PAAS freeze-dried porous polymer was fabricated by freeze-drying, with the preparation process illustrated in Figure 1. PAAS powder and PVA powder were dissolved in ultrapure water at 25 °C and 90 °C, respectively. After complete dissolution, the PVA solution (7 wt%) was mixed with isovolumetric PAAS solution (1.5 wt%). Subsequently, 50 mL of the resulting mixture was poured into a 10 × 10 cm plastic mold and frozen at −80 °C for 12 h. The sample was then thawed at room temperature for 5 h and subjected to three freeze–thaw cycles, followed by freeze-drying for 48 h. The obtained freeze-dried porous polymers were labeled as PP-1788, PP-1797, PP-1799, PP-W1, PP-W2 and PP-W3, respectively (Table 1). Before further characterization, all freeze-dried porous polymer samples were equilibrated at constant temperature and humidity for 24 h. PP-1799 and PP-W2 were alternative designations for the same sample.

### 2.3. Characterization of Freeze-Dried Porous Polymer

#### 2.3.1. SEM

The morphological study of the freeze-dried porous polymer was performed using scanning electron microscopy (SU1510 SEM, Hitachi, Tokyo, Japan) with an acceleration voltage of 10 KV. Before observation, representative cross-sections of freeze-dried porous polymer were uniformly sectioned for measurement [27].

#### 2.3.2. Porosity

The microstructure of the freeze-dried porous polymer is reflected by its porosity. The dried freeze-dried porous polymer weighed (m_1_), fully immersed in ethanol (m), and evacuated in a vacuum desiccator until no bubbles emerged. After vacuum treatment, the freeze-dried porous polymer was removed, and the container with residual ethanol was weighed (m_2_) [28]. Calculation of the porosity of the freeze-dried porous polymer according to Equation (1):

(1) $$Porosity\;(\%)=\frac{m-m_{2}-m_{1}}{m-m_{2}}\times 100$$

#### 2.3.3. Density

The density (ρ) of the freeze-dried porous polymer was determined using a geometric method (based on cylindrical dimensions) adapted from MIRMOEINI et al. [29]. The volume (V) was measured using a digital caliper, while the mass (m) was measured using a high-precision digital balance. Calculation of the density of the freeze-dried porous polymer according to Equation (2):

(2) $$\rho \left(mg/{cm}^{3}\right)=\frac{m}{v}$$

#### 2.3.4. X-Ray Diffraction (XRD)

The XRD images for the freeze-dried porous polymer were obtained using X-ray diffractometry (DX-2700BH, Haoyuan, Liaoning, China) The samples were carried out at ambient temperature, with a diffraction angle (2θ) ranging from 5° to 60° and a scanning rate set at 10°/min [30].

#### 2.3.5. Fourier Transform Infrared Spectroscopy (FTIR)

The chemical structure of the freeze-dried porous polymer was characterized using an FTIR spectrometer (TENSOR 27, Bruker, Karlsruhe, Germany) equipped with an attenuated total reflectance (ATR) accessory. Spectra were recorded over the wavenumber range of 4000–600 cm^−1^ with 64 accumulated scans [31]. A background spectrum of ambient air was acquired and automatically subtracted before sample analysis.

#### 2.3.6. Thermal Gravimetric Analyzer

The thermal stability of the freeze-dried porous polymer was analyzed using a thermogravimetric analyzer (Pyris Diamond TG/DTA, Perkin Elmer, Waltham, MA, USA). Under a nitrogen (N_2_) atmosphere, samples weighing between 3 and 5 mg were heated from 30 °C to 500 °C at a heating rate of 15 °C/min.

#### 2.3.7. Water Absorption Capacity (WAC)

For the measurement of water absorption, the prepared freeze-dried porous polymer was placed in a desiccator for 48 h until a constant weight was achieved. The water absorption capability of freeze-dried porous polymer was measured by immersing a pre-weighed dry sample in distilled water and weighing it at different specific times. To determine the initial mass, a 0.1 g square sample was placed in a sealed container along with 20 mL of distilled water and weighed. The samples were periodically taken out of the liquid and weighed after removing the excess liquid. Measurements were taken until the samples were equilibrated and the total weight gain was calculated. The excess surface water was removed with filter paper before weighing. This analysis was carried out in triplicate and the mean value was provided [32,33]. The water absorption capacity was calculated using Equation (3):

(3) $$Water\;absorption\;capacity\;(\%)=\frac{W_{1}-W_{2}}{W_{2}}\times 100$$

where W_1_ is the weight of the sample at a specific time, and W_2_ is the weight of the dry sample.

#### 2.3.8. Water Absorption Dynamic Curve

Water absorption was measured at 0.5, 1.0, 1.5, 2.5, 5.0, and 24 h to calculate the water absorption rate. According to the method of Zhao et al. [34], the kinetic data for water sorption were analyzed using the pseudo-first order Equation (4) and pseudo-second order models Equation (5) to reveal the underlying mechanism.

(4) $$L_{n}(\frac{q_{e}}{q_{e}-q_{t}})=K_{1}t$$

(5) $$\frac{t}{q_{t}}=\frac{1}{K_{2}q_{e}^{2}}+\frac{t}{q_{e}}$$

where q_e_ (g/g) was the equilibrium water sorption rate, q_t_ (g/g) was the water sorption rate under different time points, K_1_ and K_2_ were the rate constants for water absorption kinetics in the pseudo-first-order model and the second-order model, respectively.

### 2.4. Statistical Analysis

Origin software 2024 (Stat-Ease Inc., Minneapolis, MN, USA) was used to analyze the results and draw figures. All values are expressed as mean ± standard deviation (SD) from three independent biological replicates. Statistical significance was determined by one-way ANOVA using SPSS Statistics (v26.0), and significant differences (*p* < 0.05) among different treatments were verified using Duncan’s method.

## 3. Results

### 3.1. Morphology of Freeze-Dried Porous Polymer

**Figure 2:**
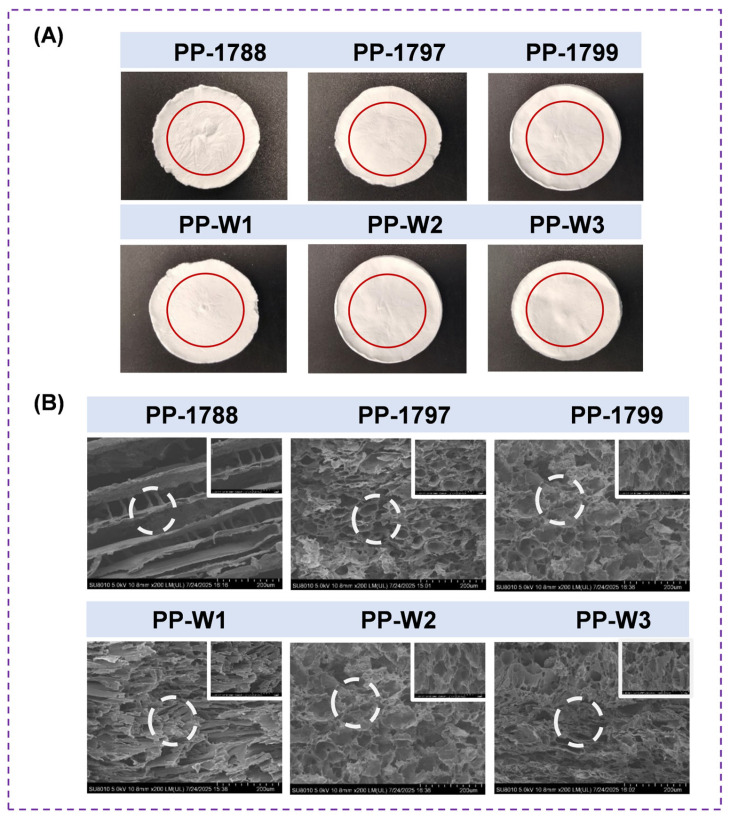
Apparent morphologies (**A**) and scanning electron microscopy cross-section (**B**) of PVA freeze-dried porous polymer with different degrees of hydrolysis and molecular weights.

The microstructure, apparent morphology and pore size distribution of polyvinyl alcohol freeze-dried porous polymer with different degrees of hydrolysis and molecular weight were shown in Figure 2. The appearance of all prepared freeze-dried porous polymers is white. Of all the samples in the same area, PP-1788 has the most abundant surface wrinkles (Figure 2A). At the same molecular weight (Figure 2B), the PP-1788 freeze-dried porous polymer has a loose stacking structure with few interlayer-connecting skeletons and cannot self-assemble to form a stable three-dimensional cellular structure. The pore structure of freeze-dried porous polymer increases with increasing PVA molecular weight at a fixed degree of hydrolysis. However, beyond a certain threshold, the pore structure becomes smaller.

### 3.2. Porosity and Density

**Figure 3:**
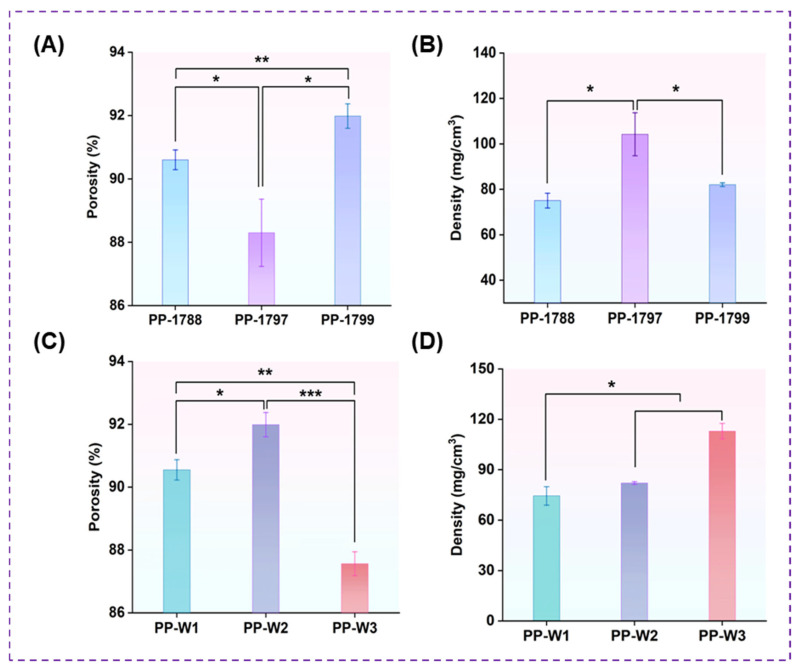
Porosity (**A**) and density (**B**) of PVA freeze-dried porous polymer with different degrees of hydrolysis. Porosity (**C**) and density (**D**) of PVA freeze-dried porous polymer with different molecular weights. These values serve to indicate comparative trends and are not intended as precise measures of absolute structural parameters. * *p* < 0.05, ** *p* < 0.01, and *** *p* < 0.001.

Figure 3 shows the porosity and density of freeze-dried porous polymer with different PVA degrees of hydrolysis and molecular weights. The porosities of PP-1788, PP-1797, and PP-1799 were 91 ± 0.31%, 88 ± 1.06%, and 92 ± 0.40% (Figure 3A), with corresponding densities of 75 ± 3.24 mg/cm^3^, 104 ± 9.47 mg/cm^3^, and 82 ± 0.81 mg/cm^3^, respectively (Figure 3B). The density of PP-1797 is significantly higher than that of PP-1788 and PP-1799 (*p* < 0.05). The porosities of PP-W1, PP-W2, and PP-W3 were 91± 0.32%, 92 ± 0.38% and 88 ± 0.38% (Figure 3C), with corresponding densities of 75 ± 5.49 mg/cm^3^, 82 ± 0.81 mg/cm^3^ and 113 ± 4.60 mg/cm^3^, respectively (Figure 3D). The higher molecular weight freeze-dried porous polymer PP-W3 exhibits significantly lower porosity than PP-W1 and PP-W2 (*p* < 0.05).

### 3.3. XRD

**Figure 4:**
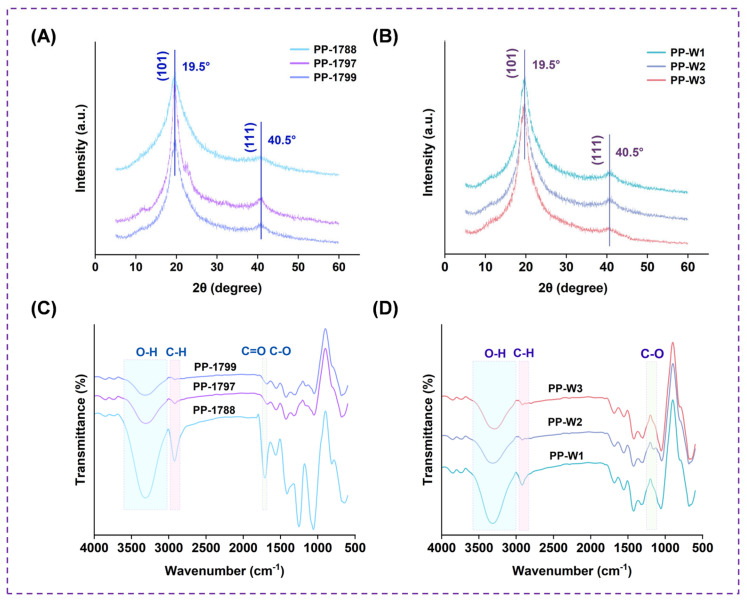
XRD patterns of PVA freeze-dried porous polymer with different degrees of hydrolysis (**A**) and molecular weights (**B**). FTIR spectra of PVA freeze-dried porous polymer with different degrees of hydrolysis (**C**) and molecular weights (**D**).

The effect of polyvinyl alcohol with different degrees of hydrolysis and molecular weights on the crystal structure of superabsorbent freeze-dried porous polymer was investigated by XRD. As shown in Figure 4A,B, a diffraction peak with relatively high intensity and large full width at half maximum appears at 2θ = 19.5° [35]. Since PAAS is amorphous and shows no diffraction peaks, the observed characteristic peak can be solely attributed to the (101) crystal plane of PVA. Another diffraction peak of PVA is observed at 2θ = 40.5°, which is assigned to the (111) crystal plane.

### 3.4. FTIR

Figure 4C,D show the FTIR spectra of freeze-dried porous polymer with different PVA hydrolyzation degrees and molecular weights. Absorption peaks were observed at 3600–3000 cm^−1^, 2900–2840 cm^−1^, and 1750–1680 cm^−1^ (Figure 4C), corresponding to O–H stretching, C–H stretching, C=O and C–O stretching, respectively [36]. Under conditions of the identical degree of hydrolysis but different molecular weights (Figure 4D), the absorption peaks observed at 1110–1250 cm^−1^ were mainly C–O groups. The absorption band observed at 1750–1680 cm^−1^ is ascribed to the carbonyl stretch of residual acetate groups originating from the incomplete hydrolysis of PVA [24].

### 3.5. Thermal Gravimetric Analyzer

**Figure 5:**
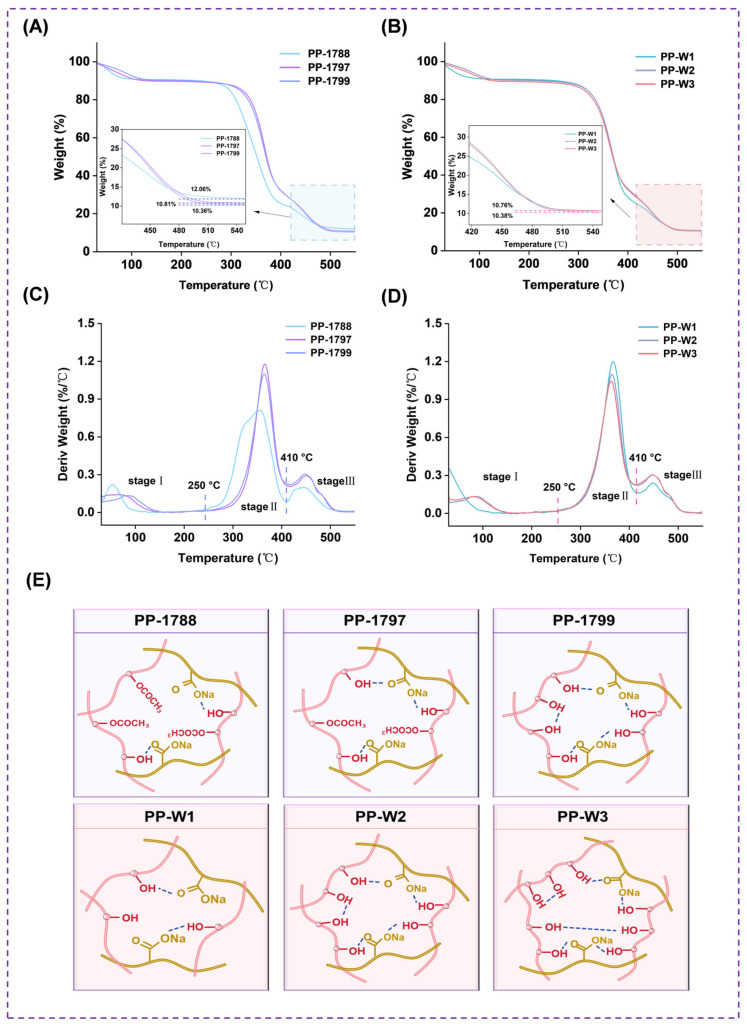
Thermodynamic analyses: TG of PVA freeze-dried porous polymer with different degrees of hydrolysis (**A**) and molecular weights (**B**). DTG of PVA freeze-dried porous polymer with different degrees of hydrolysis (**C**) and molecular weights (**D**). Molecular cross-linking diagram (**E**) of PVA freeze-dried porous polymer with different degrees of hydrolysis and molecular weights.

Thermal gravimetric analysis was employed to evaluate the thermal stability of freeze-dried porous polymer samples, as shown in Figure 5. The TG curves (Figure 5A,B) for freeze-dried porous polymer across all groups all exhibit the same three-stage mass loss. Initial thermal degradation of freeze-dried porous polymer occurs between 30~250 °C. This stage is primarily due to the loss of a portion of free water in the samples and the degradation of loosely structured small molecules [37], accounting for approximately 9% mass loss. The thermal degradation process of PVA-PAAS freeze-dried porous polymer primarily occurs in the second stage (250~410 °C), where a significant weight loss of approximately 66% is observed. The thermal weight loss observed at this stage is primarily due to the severe cracking of the three-dimensional network structure of freeze-dried porous polymer. PVA backbone undergoes chain breakage, and the hydroxyl side groups subsequently undergo elimination reactions. At elevated temperatures, the intermolecular hydrogen bonds that sustain the network were disrupted, triggering a complete structural collapse. During the subsequent third stage (410~550 °C), the residual carbonaceous intermediates and crosslinked structures undergo further deep degradation and carbonization until the formation of thermally stable residues. Based on DTG analysis of samples with identical molecular weights (Figure 5C), PP-1788 exhibited a maximum thermal degradation temperature of 354 °C, while both PP-1797 and PP-1799 reached 365 °C. At a fixed degree of hydrolysis (Figure 5D), the maximum thermal degradation temperatures of PP-W1, PP-W2 and PP-W3 were 367 °C, 365 °C and 362 °C, respectively. As shown in Figure 5E, schematic illustration of the molecular structure entanglement in PVA/PAAS freeze-dried porous polymer with varying degrees of hydrolysis and molecular weights.

### 3.6. WAC

**Figure 6:**
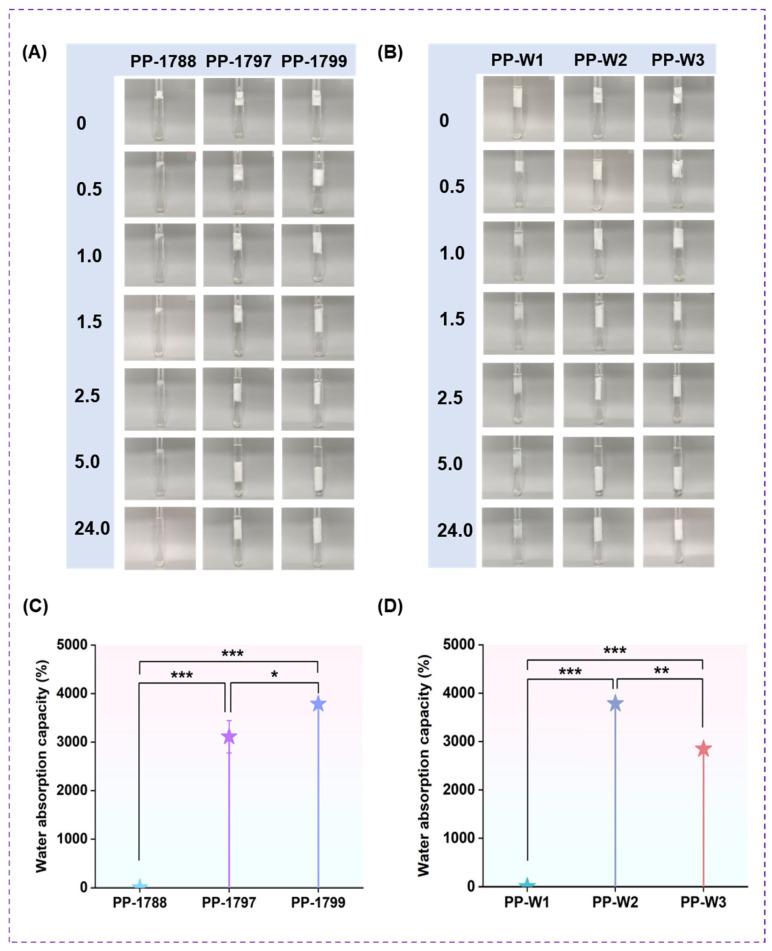
Morphological changes in the water absorption of PVA freeze-dried porous polymer with different degrees of hydrolysis (**A**) and molecular weights (**B**) at various time points. Water absorption capacity of PVA freeze-dried porous polymer with different degrees of hydrolysis (**C**) and molecular weights (**D**). * *p* < 0.05, ** *p* < 0.01, and *** *p* < 0.001.

Figure 6 illustrates the effect of polyvinyl alcohol with varying degrees of hydrolysis and molecular weights on the WAC of a highly absorbent freeze-dried porous polymer. The apparent morphological changes in freeze-dried porous polymer during water absorption within 24 h are shown in Figure 6A,B. At an 88% degree of hydrolysis, the freeze-dried porous polymer dissolved after 24 h, whereas at a molecular weight of W1, the freeze-dried porous polymer structure fragmented within the same period. As shown in Figure 6C,D, the water absorption capacity exhibited significant changes with an increase in the degree of PVA hydrolysis and the length of the molecular chain. The WAC of the freeze-dried porous polymer increases continuously with the rise in PVA hydrolysis degree. As the molecular weight increases, the WAC of the freeze-dried porous polymer initially increases and then decreases. The values of WAC for PP-1797, PP-1799 (PP-W2) and PP-W3 were 3109 ± 334%, 3785 ± 57% and 2845 ± 102% respectively.

### 3.7. Water Absorption Kinetics

**Figure 7:**
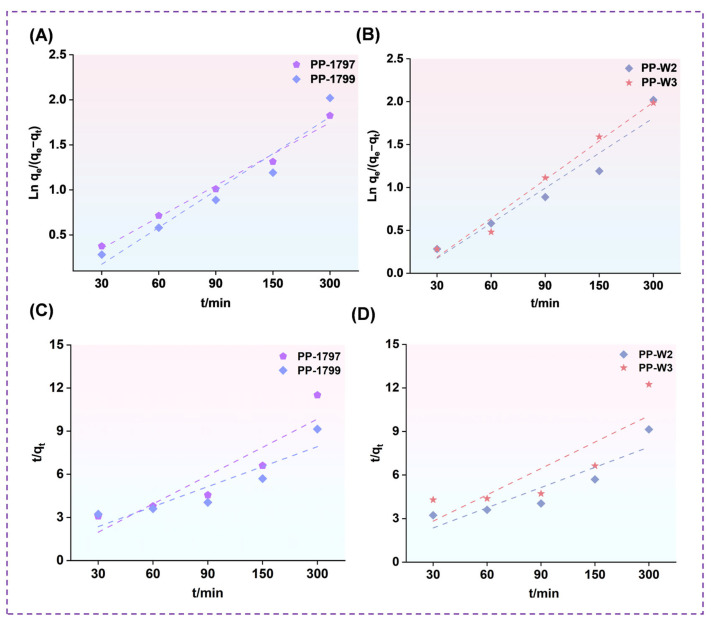
Pseudo-first-order of PVA freeze-dried porous polymer with different degrees of hydrolysis (**A**) and molecular weights (**B**). Pseudo-second-order of PVA freeze-dried porous polymer with different degrees of hydrolysis (**C**) and molecular weights (**D**).

The absorption kinetics of water were studied based on pseudo-first order (Figure 7A,B) and pseudo-second order (Figure 7C,D) models to evaluate the freeze-dried porous polymer adsorption mechanism. The specific kinetic parameters were listed in Table 2. The values of R^2^ for the pseudo-first order and pseudo-second-order models of PP-1797 and PP-1799 were 0.98, 0.92, 0.78, and 0.76, respectively, while those for PP-W2 and PP-W3 were 0.92, 0.98, 0.76, and 0.63, respectively.

## 4. Discussion

This study optimized the structure of the PAAS freeze-dried porous polymer by adjusting the degree of hydrolysis and molecular weight of PVA. Morphological characterization of the freeze-dried porous polymer shows an increase in PVA degrees of hydrolysis. PVA with a high degree of hydrolysis provides an abundance of hydroxyl functional groups and a small amount of acetate groups. The formation of many hydrogen bonds with carboxyl groups on the sodium polyacrylate chain reduces the number of free hydroxyls groups and significantly increases the crosslinking density [38]. The strong hydrogen bond interaction and molecular entanglement occur between PAAS and PVA, thus forming a dense and uniform cell structure [39]. Furthermore, a shorter molecular weight means fewer hydroxyl groups, which in turn suppresses pore formation and weakens the freeze-dried porous polymer. Simultaneously, excessively strong hydrogen bonding leads to overly tight intermolecular entanglements, further restricting pore formation. The pore structure of the freeze-dried porous polymer was optimized by adjusting the molecular parameters of PVA, thereby influencing its water absorption properties. As described above, this study confirms the excellent compatibility and interaction between sodium polyacrylate and PVA molecular chains. This three-dimensional multi-porous cellular structure enhances the material’s water absorption capacity. Although PP-1788 freeze-dried porous polymer possesses high porosity, its low PVA hydrolysis degree results in insufficient intermolecular hydrogen bond crosslinking density, preventing the formation of a stable microporous structure. This structural weakness prevents the formation of a stable microporous structure and causes collapse and dissolution upon water contact. The inverse relationship between porosity and density in freeze-dried porous polymer produces materials that are lightweight and have a low bulk density. Employing a high degree of hydrolysis of PVA and optimizing its molecular weight is crucial for obtaining a freeze-dried porous polymer with excellent structural integrity and water stability.

The crystal structure shows that as the degree of hydrolysis increases, the intensity of its characteristic diffraction peaks at 19.5° markedly enhances. The higher hydroxyl content promotes hydrogen bonding interactions, which in turn facilitate the ordered arrangement and crystallization of the network. However, at the same degree of hydrolysis, changes in molecular weight did not result in significant alterations in diffraction peak intensity. This indicates that within the molecular weight range studied here, the crystallization process was primarily governed by local interactions between segments, while variations in overall chain length were insufficient to influence crystallization capability. Infrared spectroscopy results show that the presence of a broad absorption band in the region of 3600–3000 cm^−1^ indicates intermolecular and intramolecular interactions in hydroxyl (OH) crosslinking of freeze-dried porous polymer. These results indicate that PVA-PVA and PVA-PAAS interactions act simultaneously [26]. Under identical molecular weight conditions, the residual acetate groups in freeze-dried porous polymer with lower hydrolysis degrees did not react with PAAS, leading to a relatively higher carbonyl content and fewer effective hydrogen bonds. Simultaneously, under conditions of complete PVA hydrolysis, shorter molecular chains may exhibit stronger adsorption peaks in the spectrum due to reduced interchain entanglement. The consistent positions of the C=H peaks across all treatment groups further confirm the occurrence of a reaction between hydroxyl and carboxyl groups. Freeze-dried porous polymer with shorter molecular chains may exhibit stronger absorption peaks due to reduced entanglement between the chains. The creation of the three-dimensional network structure of freeze-dried porous polymer is mostly impacted by hydrogen bonding. The primary sources of this force were ion–dipole interactions (PVA–PAAS) and hydroxyl–hydroxyl interactions (PVA–PVA). These two types of hydrogen bonds work together to construct and stabilize the porous structure of the freeze-dried polymer. The thermal stability results showed that a lower PVA molecular weight, with shorter chain segments, resulted in a reduced total number of available OH groups. Although some COONa groups remain unreacted, the chain can fully extend and form effective, uniform hydrogen-bond crosslinks with those available on the sodium polyacrylate. An increase in PVA molecular weight significantly raises the number of hydroxyl groups per chain. Consequently, even if the degree of reaction with COONa is higher, the system nevertheless ends up with more uncrosslinked “free” hydroxyl groups. These free hydroxyl groups serve as weak points for thermal degradation, resulting in a decline in the material’s overall thermal stability.

The WAC and stability of a freeze-dried porous polymer are among the key performance indicators. The dissolution of PP-1788 in water was attributed to its high content of residual acetate groups, which results in a lower availability of hydroxyl functional groups for cross-linking. Hydroxyl groups play a crucial role in forming intermolecular hydrogen bonds and constructing physical crosslinking networks. A decrease in hydroxyl groups weakens hydrogen bonding, thereby reducing crosslinking density. This compromises the network integrity and leads to material dissolution in water. Shorter molecular chains limit the effective entanglement within the three-dimensional network, resulting in insufficient freeze-dried porous polymer strength. The resulting freeze-dried porous polymer structure is unstable and prone to cracking under the stress of water absorption and expansion. As a result, the water absorption rate could not be accurately determined. The greater the degree of hydrolysis, the higher the density of hydroxyl groups on the molecular chain. These hydroxyl groups form stronger intermolecular hydrogen bonds with the carboxyl groups on the sodium polyacrylate chain and enhance the intramolecular hydrogen bonding between PVA chains. Consequently, the intermolecular entanglement becomes more pronounced [39]. This synergistic cross-linking effect creates a more robust, integrated three-dimensional network. This gives the freeze-dried porous polymer excellent water stability and a high absorption capacity. The short molecular chains limit the orderly arrangement of the polymers, resulting in a tendency toward irregular entanglement [35]. Sodium polyacrylate contains a high concentration of hydrated sodium ions. When the freeze-dried, porous polymer is immersed in water, these ions attract numerous water molecules, while diffusion out of the network is restricted by the immobilized polyanions. External water molecules attempt to enter the network to dilute the concentration of ions, thereby achieving equilibrium between the interior and exterior. The freeze-dried porous polymer’s water absorption is further facilitated by osmotic pressure driven by concentration gradients. Based on the R^2^ coefficient, the pseudo-first-order model was higher than that of the pseudo-second-order model, and these values were closer to unity. It can be concluded that the pseudo-first-order model more accurately describes the water absorption kinetics of the freeze-dried porous polymer. These findings suggest that the pseudo-first-order model is a more accurate representation of the kinetics of water absorption in freeze-dried porous polymer. This suitability can be attributed not only to the abundance of hydrophilic groups but also to hydrogen bonding between biopolymer chains. Furthermore, the adsorption process is primarily governed by physical interactions. Specifically, freeze-dried porous polymer prepared with a degree of hydrolysis of 99% and a molecular weight of 7200–8100 exhibited the highest values of WAC (Supplementary Materials, Video S1).

## 5. Conclusions

The effects of polyvinyl alcohol (PVA) hydrolytic degree and molecular weight on its crosslinking density with sodium polyacrylate (PAAS) were systematically investigated. The structural changes in the freeze-dried porous polymer were elucidated by analyzing the effects of this regulatory approach. Hydrogen bonding and molecular entanglement between polymer chains were responsible for enhanced water stability and a three-dimensional cellular network in the freeze-dried porous polymer. This resultant structure, in turn, is the critical determinant of its WAC. Increasing the degree of hydrolysis of PVA led to consistent enhancements in the freeze-dried porous polymer’s water stability, water absorption rate, and thermal stability. Additionally, PVA with a higher molecular weight exhibits improved stability but also significantly reduces the WAC of the freeze-dried porous polymer. Excessive cross-linking severely diminishes the WAC, demonstrating a detrimental effect on the properties of freeze-dried porous polymer. In summary, this study has identified the key parameters for regulating the water-stable structure of PVA freeze-dried porous materials. The study also provides data support for their related applications.

## Figures and Tables

**Table 1:** The molecular weight and degree of hydrolysis of different PVA/PAAS samples.

Sample	Molecular Weight	Degree of Hydrolysis
PP-1788	72,000~81,000	87~89%
PP-1797	72,000~81,000	96~98%
PP-1799	72,000~81,000	98~99%
PP-W1	31,000~50,000	98~99%
PP-W2	72,000~81,000	98~99%
PP-W3	190,000	98~99%

**Table 2:** Kinetic tests of PVA freeze-dried porous polymer with different degrees of hydrolysis and molecular weights.

Samples	Model	Curve-Fitted Equation	R^2^	Kinetic Data (g/g/min)
PP-1797	Pseudo-first-order kinetic	y= 0.350x − 0.001	0.98	k_1_ 0.35
	Pseudo-second-order kinetic	y = 1.967x + 0.002	0.78	k_2_ 1.97
PP-1799\PP-W2	Pseudo-first-order kinetic	y = 0.409x − 0.234	0.92	k_1_ 0.41
	Pseudo-second-order kinetic	y = 1.392x + 0.966	0.76	k_2_ 1.40
PP-W3	Pseudo-first-order kinetic	y = 0.452x − 0.265	0.98	k_1_ 0.45
	Pseudo-second-order kinetic	y = 1.814x + 1.007	0.63	k_2_ 1.81

## Data Availability

The data reported in this study can be obtained by submitting a request to the corresponding author. Due to privacy considerations, these data are not publicly accessible.

## Supplementary Materials

The following supporting information can be downloaded at: https://www.mdpi.com/article/10.3390/bioengineering13030259/s1, Video S1: The aerogel of PP-1799 liquid absorption process.

## Abbreviations

The following abbreviations are used in this manuscript:

PVA	Polyvinyl alcohol
PAAS	Sodium polyacrylate
PP-1788	Mw (72,000–81,000) hydrolysis (87–89%)
PP-1797	Mw (72,000–81,000) hydrolysis (96–98%)
PP-1799	Mw (72,000–81,000) hydrolysis (98–99%)
PP-W1	Hydrolysis (98–99%) Mw (31,000–50,000)
PP-W2	Hydrolysis (98–99%) Mw (72,000–81,000
PP-W3	Hydrolysis (98–99%) Mw (190,000)
